# Associations of cardiorespiratory fitness and muscular fitness with plasma levels of endocannabinoids and their analogues in adults with diagnosed depression: SONRIE study

**DOI:** 10.1007/s00406-025-02032-w

**Published:** 2025-06-05

**Authors:** Manuel Ruiz-Muñoz, Sonia Ortega-Gómez, Maria del Mar Espinosa-Nogales, Eulalio Valmisa-Gómez de Lara, Miguel Ángel Rosety-Rodríguez, Vanesa España-Romero

**Affiliations:** 1 MOVE-IT Research group, Department of Physical Education, Faculty of Education Sciences, University of Cadiz, Cadiz, Spain; 2 Instituto de Investigación e Innovación Biomédica de Cádiz (INiBICA), Cádiz, Spain; 3 Mental Health Service, Puerto Real University Hospital, Cadiz, Spain; 4 C-HIPPER Climbing Research Association, Cadiz, Spain

**Keywords:** Depression, Endocannabinoid system, Physical fitness, Cardiorespiratory fitness, Muscular fitness, Cross-sectional study

## Abstract

**Background:**

Depression is a leading cause of global disability, affecting approximately 280 million individuals worldwide. Emerging evidence suggests that both physical fitness and endocannabinoids system play significant roles in the pathophysiology and management of depressive disorders.

**Purpose:**

To examine the associations between physical fitness, in terms of cardiorespiratory fitness (CRF) and muscular fitness (MF), with plasma levels of endocannabinoids (eCBs) and their analogues, in adults diagnosed with depression.

**Methods:**

The study involved 80 adults (ages 25–65) with a psychiatric diagnosis of mild-to-moderate depression based on the International Classification of Diseases 10th Revision (ICD-10) criteria. Physical fitness was assessed through tests measuring CRF (6-Minute Walk Test) and MF (Handgrip, Arm Curl, Chair Stand, Standing Long Jump Test). Fasting plasma levels of eCBs, such as anandamide (AEA) and 2-arachidonoyl glycerol (2-AG), and their analogues were measured using liquid chromatography-tandem mass spectrometry. Linear regression analyses were conducted or the associations between fitness variables and plasma levels of eCBs and their analogues using three models.

**Results:**

CRF and jump performance were inversely associated with plasma levels of the eCBs AEA (β range: − 0.302 to − 0.237 for CRF, − 0.315 to − 0.370 for jump; All *p* < 0.05) and 2-AG **(**β range: − 0.308 to − 0.326 across all models). eCBs analogues correlated negatively with CRF [2-linoleoylglycerol, 2-oleoylglycerol, docosatetraenoylethanolamide (DEA)] and jump (linoleylethanolamine, stearoylethanolamine, DEA); findings persisted after lean mass normalization.

**Conclusion:**

Our findings indicated that higher physical fitness was associated with lower plasma levels of eCBs and their analogues.

**Supplementary Information:**

The online version contains supplementary material available at 10.1007/s00406-025-02032-w.

## Introduction

Depressive disorders are characterized by sustained negative affect and diminished positive affect [1]. Diagnostic criteria include persistent sadness and loss of interest in normally enjoyable activities, accompanied by an inability to perform daily tasks, for at least two weeks [2–4]. Currently, depression is among the leading cause of disability worldwide [5, 6], affecting approximately 280 million individuals [7]; and is projected to rank first in global disease burden by 2030 [5, 8].

Scientific evidence suggests that a high level of physical fitness positively influences mental health status [9, 10]. Epidemiological studies have demonstrated that cardiorespiratory fitness (CRF) is inversely associated with psychological syndromes such as depression, anxiety and panic attacks [10–12]. Moreover, greater muscular fitness (MF) shows positive effects on individuals physical and mental health [13]. These beneficial effects of physical fitness extend to molecular and neurobiological mechanisms, including modulation of the endocannabinoid system (ES) [14], which plays a crucial role in mood and stress regulation​ [15].

Emerging evidence highlights the significance of the ES in depression [16]. The ES is a broad neuromodulatory network that influences cognitive and emotional behaviors, neurogenesis, and neurotrophin levels, such as brain-derived neurotrophic factor [16–18]. The primary endocannabinoids (eCBs), anandamide (AEA) and 2-arachidonoylglycerol (2-AG), are synthesized on demand in both central and peripheral tissues [19]. When these eCBs bind to cannabinoid receptors, they influence various physiological processes. Cannabinoid type 1 (CB1) receptors are primarily found in brain regions, while cannabinoid type 2 (CB2) receptors are mainly located in immune tissues and inflammatory cells, with lower densities in the brain [20]. Through these interactions, eCBs help regulate mood and stress response [16–18].

Beyond the primary eCBs, the ES also includes structurally related, lipid-derived molecules such as oleoylethanolamide (OEA), 2-linoleoylglycerol (2-LG), 2-oleoylglycerol (2-OG), docosatetraenoylethanolamide (DEA), docosahexaenoylethanolamide (DHEA), linoleylethanolamine (LEA), and stearoylethanolamine (SEA) [19]. Although these analogues do not interact directly with CB1 or CB2 receptors, they influence mental health via CB receptor-independent mechanisms. Specifically, they activate peroxisome proliferator-activated receptors (PPAR-α and PPAR-γ) [19], modulate transient receptor potential vanilloid-1 (TRPV1) channels [21], and inhibit fatty acid amide hydrolase (FAAH), the enzyme responsible for AEA degradation. By inhibiting FAAH, these molecules enhance AEA signaling [22], contributing to inflammation control, neuroprotection, and the regulation of mood and stress responses, which are critical in the pathophysiology of depression [19, 21, 22]. Notably, depressed individuals have been reported to exhibit lower plasma levels of eCBs and their analogues, supporting the hypothesis of an endocannabinoid deficiency in affective disorders [20, 23].

Numerous studies have examined the effects of acute or chronic exercise on plasma levels of eCBs and their analogues [21, 24–26]; however, to our knowledge, no studies have investigated the association between physical fitness and plasma levels of these molecules in individuals with depression. Given that physical fitness—quantified here as CRF and MF—reflects long-term adaptations to regular activity, we hypothesize that individuals with depression who exhibit higher fitness levels will have lower plasma levels of eCBs and their analogues. Consequently, the purpose of our study was to examine these associations in individuals diagnosed with depression.

## Methods

### Study design

This study is part of the SONRIE study, a randomized controlled trial (RCT) registered at ClinicalTrials.gov (Identifier: NCT05849792). It adheres to the 2013 Declaration of Helsinki, approved by the Andalusian Biomedical Research Ethics Portal (1875-N-18 CEI/ Cádiz). All participants provided written informed consent prior to inclusion.

Eligibility criteria included adults aged 25 to 65 years due to the highest prevalence of depression in this demographic in Andalusia [27], with a psychiatric diagnosis of mild-to-moderate depression according to International Classification of Diseases 10th Revision (ICD-10) criteria [28]. We limited our study to individuals with mild-to-moderate depression to ensure participants safety and to maintain a homogeneous sample, as physical exercise has been shown to be an effective intervention in this population [29]. Project investigators subsequently verified that participants met the remaining criteria, including the ability to engage in physical activity without restrictions. Exclusion criteria included a diagnosis of major depression, the presence of acute or terminal illness, a history of cerebral infarction, epilepsy, or brain cancer, and unstable cardiovascular disease or other medical conditions that could interfere with participation in physical exercises.

### Participants

The SONRIE study engaged 132 individuals diagnosed with depression by a psychiatrist from the Mental Health Services of Puerto Real (Cádiz). All participants were in a stable, post-acute phase of depression, and were recruited through a detailed outreach process that included community presentations and social media campaigns. Of these, 120 attended an information meeting and 94 requested the pre-screening questionnaire. After assessing for eligibility, 84 individuals met the pre-screening criteria and 80 were eligible for final assessment.

### Measures

#### Interview measures

Sociodemographic characteristics were collected using a self-designed developed structured questionnaire. The data captured included age, antidepressant usage, economic stress, smoking and alcohol intake, educational level, and marital status. Additionally, information on comorbidities and the impact of bodily pain on daily functioning was obtained by asking whether participants had ever been diagnosed with conditions such as cardiovascular diseases, cancer, or diabetes (recorded as ‘yes’ or ‘no’). Responses for antidepressant usage and economic stress were binary (“yes” or “no” for medication usage; “easy” or “difficult” for economic stress). Smoking status and alcohol intake were classified into three categories: “current”, “former” or “never”. Educational level was dichotomized into “up to primary education” or “higher education”, and marital status was coded as “single” or “in a relationship”. The persistence of depression was recorded as the number of years since the diagnosis.

#### Physical appraisal

##### Body composition

Body composition was assessed using a multifrequency bioimpedance analyzer (TANITA-MC780MA) and a stadiometer (Type SECA 225). Specifically, body weight (kg), height (cm), body mass index (BMI, kg/m²), fat mass (kg), fat mass percentage, lean mass (kg), and lean mass percentage variables were measured. Height was measured in the Frankfurt plane, and BMI was calculated using the recorded weight and height values.

##### Plasma levels of endocannabinoids and endocannabinoids analogues

After an overnight fast, blood sample were collected from each participant between 08:00 and 09:00 h. through venepuncture into K2-EDTA tubes. The plasma was then immediately separated from these samples in a centrifuge pre-cooled to 4ºC, operating at 1700 G for 15 min. This process commenced within 10 min post-extraction to ensure sample integrity. To stabilize the plasma, and prior to freezing, 8 µL of orlistat (250 µg/mL in ethanol) were added to each cryotube designed for ES analyses. After centrifugation, 1 mL of plasma was aliquoted into these cryotubes, which were then stored at -80ºC until analysis. We quantified eCBs (2-AG and AEA) and their analogues (2-LG, 2-OG, OEA, DEA, DHEA, LEA, and SEA) following a previously validated method [30]. Briefly, aliquots of 0.5 mL of plasma were transferred to 12 mL glass tubes, spiked with deuterated internal standards (AEA-d4, DHEA -d4, LEA-d4, OEA-d4, 2-AG-d5, and 2-OG-d5), diluted with 0.1 M ammonium acetate buffer (pH 4.0), and extracted with tert-butyl methyl ether. The dry organic extracts were reconstituted in 100 µL of a mixture water: acetonitrile (10:90, v/v) with 0.1% formic acid (v/v) and transferred to HPLC vials. A Waters Acquity UPLC system with a Xevo TQ-Smicro Mass Spectrometry detector was used for the analysis. Chromatographic separation was performed with a Waters BEH-C18 column (2.1 × 100 mm, 1.8 μm particle size) maintained at 55 °C with a mobile phase flow rate of 0.4 mL/min. The composition of the mobile phase was: A: 0.01% (v/v) formic acid in water and B: 0.01% (v/v) formic acid in acetonitrile. The mass spectrometry analysis was performed on the multiple reaction monitoring mode (MRM). Quantification was performed absolutely by isotope dilution. Quality control samples were injected across four batches, yielding RSDs of 7.0–10.7%. The deuterated internal standards were obtained from Cayman Chemical (Ann Arbor, MI, USA) and Toronto Research Chemicals (Ontario, Canada), and solvents were from Merck (Darmstadt, Germany). Plasma concentrations of 2-AG and AEA were further compared against normative values reported by Hillard et al. [31] to provide additional context.

##### Health related physical fitness

**Cardiorespiratory fitness (CRF)** was evaluated using the 6-Minute Walking Test (6MWT). Participants were instructed to walk as fast as possible for six minutes along a flat, 60-meters circuit, aiming to cover the maximum distance possible [32]. The total distance covered in a single trial was recorded in meters for further analyses. Expected 6MWT performance values were calculated using the predictive equations proposed by Enright and Sherrill [33].

**Muscular fitness (MF)** Upper-limbs strength was assessed using the arm curl test, in which participants performed seated arm flexion-extension movements with a specified weight (2.3 kg for women and 3.6 kg for men), completing one 30-seconds trial per arm [34, 35]. The number of repetitions for each arm was recorded, then combined for analysis. Handgrip strength was measured with a digital dynamometer (TKK 5101 Grip-D), tested sequentially in the right and left hands, and adjusted according to each participant hand size [36]. Each hand was tested twice, and the highest value (in kilograms) from each hand was noted, with the mean of these maximum values used for analysis. Expected normative values stratified by sex and age were derived from international reference data reported by Tomkinson et al. [37].

Lower-limbs strength was evaluated using two tests. The chair stand test required participants to rise from and return to a seated position, arm crossed over the chest, as many times as possible within 30 s [38]. The total count of these repetitions was recorded. The standing long jump test was also administered to participants. They performed two maximal forward jumps from a standing start, feet together, and the greatest distance from the starting line to the nearest heel landing point was recorded in centimeters [39]. Results were compared against normative mean values for healthy adults, stratified by sex [40].

### Statistical analysis

Linear regression analyses were conducted to estimate standardized (*ß)* and unstandardized (b) coefficients, standard errors, and 95% confidence intervals (95%IC) for the associations between fitness variables and plasma levels of eCBs and their analogues. Each fitness measure (6MWT, handgrip strength, arm curl, chair stand, and standing jump tests) was entered as an independent variable in separate models, with eCBs and their analogues concentrations as dependent variables. The Mallow Cp criterion [41] was used to identify optimal predictor variables in the regression models estimating associations for plasma levels of eCBs and their analogues with each physical fitness measure (CRF and MF test). Confounding variables were included based on their known relationship with the SE [19, 24, 42–44].

The analysis utilized three models. Model 1 was unadjusted. Models 2 and 3 included adjustments derived from a selection process achieving a Mallow’s Cp value of 5.00. In Model 2, adjustments were tailored to each ES biomarker: for 2-AG, 2-LG, 2-OG, DEA, and DHEA, factors such as age, BMI, and diabetes were included; for AEA, OEA, LEA, and SEA, adjustments accounted for fat mass, antidepressant medication use, and diabetes. Model 3 was identical to Model 2, except that the body composition variables (BMI or fat mass) were replaced with lean mass. Finally, we undertook a sensitivity analyses examining all associations between plasma levels of eCBs and their analogues with MF normalized to lean mass.

The assumptions of normality, linearity, and homoscedasticity were verified for all ES parameters, ensuring robustness of the regression models. Interaction effects (i.e. sex by main exposures) were considered by likelihood ratio test of nested models and by examining changes in *b* coefficients greater than 10%. No significant interactions were identified, permitting the analysis of all participants as a single group. Additionally, multicollinearity was assessed and found to be absent in all models; variance inflation factors (VIFs) were below 10, averaging close to 1, indicating no concern of collinearity [45]. Statistical analyses were performed using STATA version 14.0 (Stata Corp, College Station, TX, USA), with statistical significance set at *p* < 0.05.

## Results

A total of 80 participants (83.8% female), with a mean age of 49.0 ± 9.6 years and a mean depression duration of 1.65 years from diagnosis, were included. Plasma concentrations of 2-AG and AEA were 2.80 ± 1.69 ng/mL and 0.25 ± 0.09 ng/mL, respectively, both at the lower end of ranges reported in healthy adults [31]. Physical fitness results indicated generally lower levels across the cohort: in terms of CRF, the mean 6MWT distance (539.0 ± 79.2 m) was ~ 60% of the predicted value [33]. MF assessments revealed an average handgrip strength of 25.8 ± 9.1 kg, 17.2 ± 4.3 arm curl repetitions, 11.8 ± 2.7 chair stand repetitions, and an 89.5 ± 34.1 cm standing long jump. According to international norms, 82.7% of participants fell below the median handgrip value for their sex and age group [37], and 90.1% did not reach the mean standing long jump distance reported in a healthy reference sample [40]. Detailed descriptive characteristics are presented in Table 1.

**Table 1:** Descriptive characteristics of the study sample

	*n*	mean	SD	Minimum	Maximum
**Age**	80	49.0	9.6	26.9	65.4
**Weight (kg)**	78	75.6	17.8	36.2	132.1
**Height (cm)**	78	161.8	8.8	144.3	188.4
**BMI (Kg/m²)**	78	28.8	6.0	17.4	47.5
**Fat mass (kg)**	78	27.2	11.0	7.3	66.4
**Fat mass (%)**	78	34.9	8.1	17.9	50.3
**Lean mass (kg)**	78	48.5	9.9	28.9	81.8
**Lean mass (%)**	78	65.1	8.1	49.7	82.2
**Physical Fitness**					
**Cardiorespiratory Fitness**					
*6 min Walking Test (m)*	73	539.0	79.2	362.5	752.5
**Muscular Fitness**					
*Handgrip (kg)*	79	25.8	9.1	7.6	54.5
*Arm curl (reps)*	77	17.2	4.3	6.0	26.5
*Chair stand (reps)*	78	11.8	2.7	6.0	20.0
*Standing long jump (cm)*	68	89.5	34.1	17.0	214.0
**Endocannabinoid system (ng/mL)**					
*Anandamide*	80	0.25	0.09	0.09	0.56
*2-Arachidonoylglycerol*	78	2.80	1.69	0.73	8.29
*Oleoyethanolamide*	80	2.79	0.80	1.01	6.03
*2-Linoleoylglycerol*	77	18.31	11.44	1.1	56.4
*2-Oleoylglycerol*	77	30.76	19.11	2.3	90.8
*Docosahexanoylethanolamide*	80	0.07	0.02	0.02	0.13
*Dihexanoylethanolamide*	80	0.35	0.16	0.09	1.17
*Linoleoylethanolamide*	80	0.71	0.25	0.3	1.91
*Stearoylethanolamide*	80	1.33	0.27	0.8	2.07
	**n**	**Percentages %**			
**Sex**					
*Women*	67	83.8			
*Men*	13	16.2			
**Education Level**					
*Primary School*	67	84.8			
*Secondary School*	8	10.1			
*University studies*	4	5.1			
**Cardiovascular disease (yes)**	24	31.2			
**Cancer (yes)**	20	26.0			
**Diabetes (yes)**	4	5.13			
**Marital Status**					
*Without couple*	27	34.2			
*With couple*	52	65.8			
**Smoke**					
*Never*	31	39.2			
*Former*	20	25.3			
*Current*	28	35.4			
**Alcohol**					
*Never*	22	28.2			
*Former*	19	24.4			
*Current*	37	47.4			
**Antidepressant (yes)**	65	86.7			

SD: standard deviation

Higher performance on the 6MWT was significantly associated with lower plasma levels of 2-AG across all models (Model 1: β = -0.326, *p* = 0.005; Model 2: β = -0.253, *p* = 0.041; Model 3: β = -0.308, *p* = 0.008). Similarly, AEA levels showed a negative association with CRF (Model 1: β = -0.237, *p* = 0.044), which remained significant in Model 3 (β = -0.302, *p* = 0.006). In addition, both 2-LG and 2-OG were inverse associated with the 6MWT in Model 1, with 2-LG maintaining its significance in the adjusted models. Conversely, DEA was negatively significant only in Model 3. No significant associations were observed between the 6MWT performance and SEA, LEA, or DHEA (See Table 2**)**.

**Table 2:** Associations between cardiorespiratory fitness using the 6-minute walking test (m) and plasma endocannabinoid levels in unadjusted and adjusted models

ES	Model 1					Model 2					Model 3				
	ß	b	95% IC	SE	*P*	ß	b	95% IC	SE	*P*	ß	b	95% IC	SE	*P*
*2-AG*	**-0.326**	**-0.007**	**-0.012**,** -0.002**	**0.002**	**0.005**	**-0.253**	**-0.005**	**-0.010**,** -2.00e**^**− 4**^	**0.003**	**0.041**	**-0.308**	**-0.004**	**-0.007**,** -0.001**	**0.001**	**0.008**
*AEA*	**-0.237**	**-3.00**e^− 4^	**-6.00e**^**− 4**^, **-8.4e**^**− 6**^	**1.00**e^− 4^	**0.044**	-0.092	-1.00e^− 4^	-4.00e^− 4^, 1.00e^− 4^	1.00e^− 4^	0.384	**-0.302**	**-0.004**	**-0.007**,** -0.001**	**0.001**	**0.006**
OEA	-0.122	-0.001	-0.004, 0.001	0.001	0.302	-0.086	-0.001	-0.004, 0.002	0.002	0.481	-0.153	-0.002	-0.004, 0.001	0.001	0.003
2-LG	**-0.324**	**-0.047**	**-0.080**, **-0.014**	**0.016**	**0.006**	**-0.336**	**-0.050**	**-0.085**, **-0.013**	**0.018**	**0.009**	**-0.252**	**-0.032**	**-0.006**,** -0.2e**^**− 5**^	**0.015**	**0.038**
2-OG	**-0.271**	**-0.066**	**-0.122**, **-0.010**	**0.028**	**0.022**	-0.175	-0.043	-0.102, 0.016	0.030	0.154	-0.218	-0.003	-0.005, 0.1e^− 3^	0.001	0.062
DEA	-0.224	-0.1e^− 3^	-0.14e^− 3^, 1.95e^− 6^	0.34e^− 4^	0.057	-0.108	-0.32e^− 4^	-0.1e^− 3^, 0.4e^− 4^	0.35e^− 4^	0.360	**-0.297**	**-0.9**e^− 4^	**-0.2**e^− 3^, **-0.2**e^− 4^	**0.3**e^**− 4**^	**0.011**
LEA	-0.190	-0.001	-0.001, 0.1e^− 3^	0.001	0.107	-0.165	-0.001	-0.001, 0.3e^− 3^	0.5e^− 3^	0.195	-0.205	0.7e^− 3^	-0.001, 0.9e^− 4^	0.4e^− 3^	0.084
SEA	-0.128	-0.43e^− 3^	-0.001, 0.4e^− 3^	0.4e^− 3^	0.281	-0.040	-0.13e^− 3^	-0.9e^− 3^, 0.7e^− 3^	0.39^− 3^	0.736	-0.164	-0.001	-0.001, 0.2e^− 3^	0.4e^− 3^	0.157
*DHEA*	-0.015	-0.3e^− 4^	-0.001, 0.4e^− 3^	0.23e^− 3^	0.899	0.076	0.1e^− 3^	-0.4e^− 3^, 0.001	0.3e^− 3^	0.559	0.065	0.1e^− 3^	-0.3e^− 3^, 0.001	0.2 e^− 3^	0.586

2-AG: 2-Arachidonoylglycerol; AEA: Anandamida; OEA: Oleoylethanolamide; 2-LG: 2-Linoleoylglycerol; 2-OG: 2-Oleoylglycerol; DEA: Docosatetraenoylethanolamide; LEA: Linoleoylethanolamide; SEA: Stearoylethanolamide; DHEA: Docosahexaenoylethanolamide

Data are presented for all sample as standardized regression coefficient (β), standard error (SE), regression coefficient (b), interval coefficients (95% IC) and P values from the model

Model 1: Unadjusted; Model 2: Adjusted for age, BMI and diabetes for 2-AG, 2-LG, 2-OG, DEA and DHEA or fat mass, antidepressant medication and diabetes for AEA, OEA, LEA, and SEA); Model 3: As in Model 2, but with BMI or fat mass replaced by lean mass

**Fig. 1:**
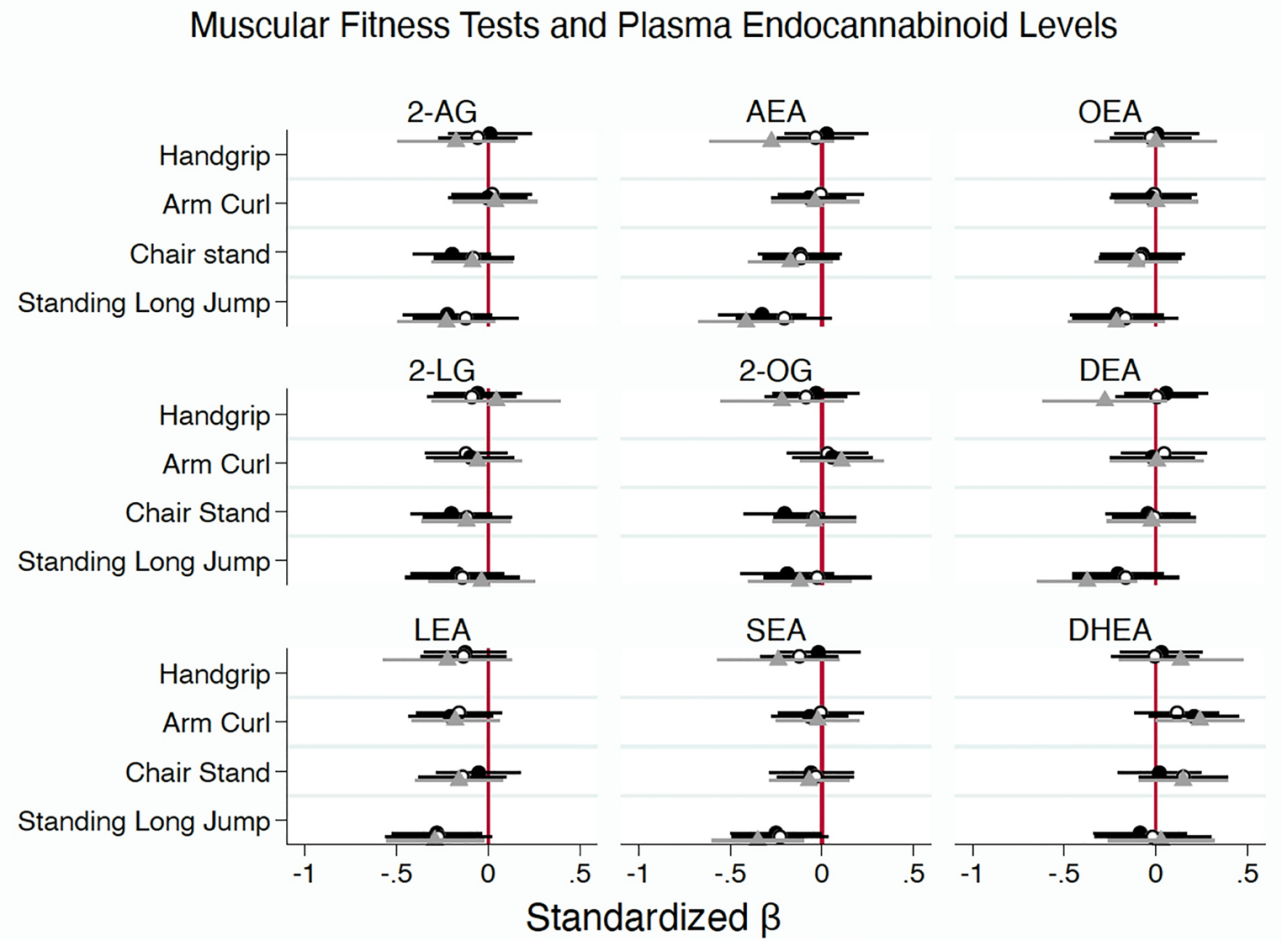
Association between Muscular Fitness Tests and Plasma Endocannabinoids Levels and their analogues in Adults with Depression. Data are presented as standardized regression coefficient (β) with 95% confidence intervals. The vertical line at 0 represents the point of no effect. Black circle (**●**): Model 1, Unadjusted; White circle (**○**): Model 2, Adjusted for age, BMI and diabetes for 2-AG, 2-LG, 2-OG, DEA and DHEA or fat mass, antidepressant medication and diabetes for AEA, OEA, LEA, and SEA); Gray triangle (▲): Model 3 As in Model 2, but with BMI or fat mass replaced by lean mass

Figure 1 illustrates the associations (β and 95% CI) between MF tests (handgrip, arm curl, chair stand, and standing long jump) and plasma levels of eCBs and their analogues. Notably, performance in the standing long jump was inversely associated with AEA levels (Model 1: β = -0.315, *p* = 0.009; Model 3: β = -0.370, *p* = 0.002). Similarly, LEA and SEA showed significant inverse associations in both unadjusted and adjusted models (LEA: Model 1: β = -0.270, *p* = 0.003; Model 3: β = -0.260, *p* = 0.035; SEA: Model 1: β = -0.240, *p* = 0.049; Model 3: β = -0.309, *p* = 0.008), while DEA reached significance only in Model 3 (β = -0.355, *p* = 0.008). In contrast, handgrip, arm curl, and chair stand tests did not demonstrate significant associations. These findings were consistent when MF was normalized by lean mass; moreover the chair stand test demonstrated a significant negative association with AEA (Model 1: β = -0.304, *p* = 0.031; Model 2: β = -0.309, *p* = 0.008) (Supplementary Table 1).

## Discussion

The present study investigated the relationship between physical fitness and plasma levels of eCBs and their analogues in adults diagnosed with depression. Our results indicate that higher performance on the 6MWT was associated with lower plasma levels of eCBs—2-AG and AEA—suggesting that these molecules may influence cardiovascular performance. In addition, significant inverse associations for certain eCBs analogues (2-LG, 2-OG, and DEA) indicate that these lipid mediators could contribute to aerobic capacity. Regarding MF, the standing long jump emerged as the test with significant associations, where elevated plasma levels of AEA, and its analogues (LEA, SEA, and DEA) were linked to poorer jump performance. Supplementary analyses, in which MF measures was normalized by lean mass, confirmed that these associations remain robust, independent of overall body composition differences. Collectively, these findings suggest a nuanced, domain-specific role of the ES, with both classical eCBs and their analogues differentially modulating aspects of physical fitness.

Two primary mechanisms could potentially explain these findings: (i) Alteration of the ES in depressive disorder. Previous research has indicated that, compared to healthy controls, individuals with chronic depression often exhibit significantly lower circulating levels of eCBs (AEA and 2-AG), with AEA levels varying according to depression severity [14, 19, 25, 46, 47]. Consistent with this, plasma levels observed in our sample fall at the lower end of the reference ranges reported for healthy individuals [31], supporting the endocannabinoid deficiency hypothesis. This hypothesis posits that diminished eCBs signaling may contribute to the pathophysiology of depressive symptoms [19]. Importantly, the levels of circulating eCBs are influenced by multiple physiological factors, including circadian rhythms, stress exposure, and metabolic demands [19]. The inverse associations observed in our study between CRF, MF, and eCBs suggest that physical fitness could play a role in modulating endocannabinoid tone, potentially as part of broader metabolic and neuromodulatory processes. Given that higher levels of physical fitness are often associated with improved metabolic regulation [48] and stress resilience [49], it is plausible that individuals with greater fitness exhibit adaptations that help maintain more stable eCB signaling. However, the precise mechanisms linking chronic fitness status to the regulation of circulating eCBs remain to be fully elucidated. Notably, while our study also assessed other biomarkers (OEA, SEA, LEA, DEA, DHEA, 2-LG, and 2-OG), their associations were less consistent across models. Given the role in metabolic regulation, inflammatory control, and energy homeostasis [19], their link to physical fitness may involve additional physiological mechanisms that warrant further investigation. (ii) Chronic stress-induced dysregulation of the sympathetic nervous system and the hypothalamic-pituitary-adrenal axis could also explain these associations [50]. Although acute stress typically increases circulating eCBs levels [19], prolonged stress exposure can have the opposite effect [51]. Improved physical fitness, particularly in terms of CRF and MF, may mitigate these adverse effects by modulating stress responses and promoting healthier endocannabinoid signaling.

Our finding align with previous studies reporting an inverse association between eCBs plasma levels (2-AG and AEA) and fitness. For instance, Koay et al. [52] demonstrated that an 80-day moderate-intensity multicomponent exercise intervention in 52 young adults reduced 2-AG levels concomitant with improved CRF. However, the literature presents divergent findings. Studies by Brellenthin et al. [24] and Jurado-Fasoli et al. [21] found no significant associations between physical activity [24] or CRF [21] and eCBs levels in non-clinical populations. Additionally, Fernández-Aranda et al. [53] reported positive associations between moderate-to-vigorous physical activity and AEA in overweight women, suggesting that the influence of exercise on circulating levels of AEA may vary depending on body composition or physical fitness status. In contrast, Moreira Antunes et al. [54] observed reduced circulating AEA levels in exercise-addicted individuals, highlighting a potential dose-response relationship between exercise intensity and endocannabinoid signaling. Adding to this complexity, Hamedinia et al. [55] reported increased post-exercise concentrations of both AEA and 2-AG in participants with improved CRF, further supporting the dynamic and context-dependent nature of the ES response to exercise. Building on these findings, Stensson et al. [26] found increased plasma AEA levels and a positive association between 2-AG and MF after a 15-week resistance exercise program in a fibromyalgia cohort. This contrasts with our results in adults with depression, highlighting how clinical characteristics (e.g., chronic pain vs. depression) may shape the endocannabinoid response to exercise.

These discrepancies likely reflect differences in study populations (e.g., healthy individuals vs. clinical populations), exercise protocols, and the timing of biomarker measurements. Our study, focused on adults with depression, provides novel insights into the role of physical fitness—particularly CRF and MF—in modulating plasma levels of eCBs and their analogues within a clinical context. Given these varied findings, future research should further explore the specific mechanisms through which CRF and MF influence ES signaling in depressed populations. Longitudinal studies examining changes in fitness alongside ES biomarkers and depressive symptoms could help establish causality and inform fitness-based interventions. Incorporating both aerobic and resistance training may offer a comprehensive approach to improve ES regulation and alleviate depressive symptoms.

Several limitations must be considered. First, no causality can be established due to the inherent limitation of all cross-sectional designs. Second, our sample is imbalanced in terms of sex (84% women) and educational attainment (85.0% with primary education, 10.0% secondary, and 5.0% university). While these demographics might limit the generalizability of our findings to populations with different sociodemographic profiles, they align with established epidemiological patterns indicating that depression is more common in women and often associated with lower educational levels [8]. Third, inherent variability in plasma eCBs levels and their analogues may constrain the clinical interpretation of our results. Fourth, physical fitness tests may be influenced by participants’ motivational states, particularly variable in a population with depression, and may also be subject to diurnal fluctuations, introducing potential bias and variability. Although practical constraints frequently necessitate the use of these measures in clinical settings, these factors may affect the precision of our assessments and the interpretation of our findings. Finally, the assessment of diabetes with a single-item question (differentiating neither type 1 nor type 2), may affect the precision of its role as a confounding factor. Despite these limitations, our study has notable strengths. It targets a clinical population and adjust for key confounders (e.g., age, BMI, fat mass, lean mass, or antidepressant use), enhancing the robustness of our findings.

## Conclusion

In summary, our findings suggested that higher cardiorespiratory fitness and better performance in the standing long jump test were inversely associated with plasma levels of endocannabinoids (2-AG, AEA) and specific analogues (2-LG, 2-OG, DEA, among others) in adults diagnosed with depression. These results suggest a potential modulatory role of physical fitness—particularly aspects of cardiorespiratory capacity and muscular fitness—on the endocannabinoid system and its network of lipid mediators, possibly through metabolic and stress-regulatory pathways. Further longitudinal studies and controlled exercise interventions are needed to confirm the directionality of these associations and their potential therapeutic potential in depression.

## Electronic supplementary material

Below is the link to the electronic supplementary material.

Supplementary Material 1
