# Supplementary material for: Associations of cardiorespiratory fitness and muscular fitness with plasma levels of endocannabinoids and their analogues in adults with diagnosed depression: SONRIE study
Source: Eur Arch Psychiatry Clin Neurosci. 2025 Jun 5;276(2):605–14. doi: 10.1007/s00406-025-02032-w (PMC12953257; doi:10.1007/s00406-025-02032-w)
Supplement: Supplementary file 1 — Supplementary Material 1 [file 406_2025_2032_MOESM1_ESM.docx]

| **Table S1:** Unadjusted and adjusted associations between muscular fitness tests normalized by lean mass, and plasma levels of endocannabinoids (and analogues). | | | | | | | | | | | | |
| --- | --- | --- | --- | --- | --- | --- | --- | --- | --- | --- | --- | --- |
| **ES** | **Muscular Fitness Tests** | | **Model 1** | | | | | **Model 2** | | | | |
|  | |  | **ß** | **b** | **(95% IC)** | **SE** | **P** | **ß** | **b** | **(95% IC)** | **SE** | **P** |
| *2-AG* | | Handgrip (kg/kg) | -1.212 | -0.096 | (-4.177, 1.752) | 1.487 | 0.418 | -0.079 | -0.999 | (-3.847, 1.849) | 1.428 | 0.785 |
|  | | Arm Curl (reps/kg) | -0.628 | -0.042 | (-4.203, 2.949) | 1.793 | 0.727 | 0.037 | 0.565 | (-3.070, 4.200) | 1.821 | 0.757 |
|  | | Chair Stand (reps/kg) | -4.645 | -0.223 | (-9.447, 0.157) | 2.408 | 0.058 | -0.118 | -2.436 | (-7.371, 2.500) | 2.473 | 0.328 |
|  | | Standing Long Jump (cm/kg) | **-0.596** | **-0.255** | **(-1.170, -0.022)** | **0.287** | **0.042** | -0.181 | -0.423 | (-1.046, 0.201) | 0.312 | 0.180 |
|  | |  |  |  |  |  |  |  |  |  |  |  |
| *AEA* | | Handgrip (kg/kg) | -0.145 | -0.201 | (-0.307, 0.017) | 0.081 | 0.083 | 0.021 | 0. 2e^-3^ | (-0.002, 0.003) | 0.001 | 0.854 |
|  | | Arm Curl (reps/kg) | -0.175 | -0.196 | (-0.381, 0.031) | 0.103 | 0.094 | -0.138 | -0.121 | (-0.329, 0.087) | 0.104 | 0.249 |
|  | | Chair Stand (reps/kg) | **-0.304** | **-0.250** | **(-0.577, -0.031)** | **0.137** | **0.031** | **-0.277** | **-0.337** | **(-0.612, -0.062)** | **0.137** | **0.017** |
|  | | Standing Long Jump (cm/kg) | **-0.056** | **-0.421** | **(-0.087, -0.026)** | **0.015** | **0.000** | **-0.378** | **-0.053** | **(-0.084, -0.022)** | **-0.016** | **0.004** |
|  | |  |  |  |  |  |  |  |  |  |  |  |
| OEA | | Handgrip (kg/kg) | -0.497 | -0.079 | (-1.950, 0.956) | 0.729 | 0.498 | 0.014 | 0.086 | (-1.309, 1.481) | 0.698 | 0.903 |
|  | | Arm Curl (reps/kg) | -0.616 | -0.079 | (-2.436, 1.205) | 0.913 | 0.502 | -0.007 | -0.052 | (-1.751, 1.646) | 0.850 | 0.951 |
|  | | Chair Stand (reps/kg) | -1.054 | -0.100 | (-3.510, 1.403) | 1.233 | 0.395 | -0.093 | -0.952 | (-3.287, 1.383) | 1.169 | 0.418 |
|  | | Standing Long Jump (cm/kg) | **-0.292** | **-0.244** | **(-0.582, -0.002)** | **0.145** | **0.049** | -0.161 | -0.194 | (-0.475, 0.085) | 0.140 | 0.169 |
|  | |  |  |  |  |  |  |  |  |  |  |  |
| 2-LG | | Handgrip (kg/kg) | -1.904 | -0.021 | (-23.325, 19.517) | 10.743 | 0.860 | 0.016 | 1.434 | (-19.673, 22.541) | 10.578 | 0.893 |
|  | | Arm Curl (reps/kg) | -4.906 | -0.047 | (-29.889, 20.078) | 12.523 | 0.696 | 0.034 | 3.534 | (-22.771, 29.839) | 13.175 | 0.789 |
|  | | Chair Stand (reps/kg) | -14.776 | -0.101 | (-49.391, 19.839) | 17.356 | 0.397 | 0.031 | 4.456 | (-32.285, 41.198) | 18.407 | 0.809 |
|  | | Standing Long Jump (cm/kg) | -1.133 | -0.069 | (-5.301, 3.036) | 2.085 | 0.589 | 0.108 | 1.760 | (-2.895, 6.415) | 2.326 | 0.452 |
|  | |  |  |  |  |  |  |  |  |  |  |  |
| 2-OG | | Handgrip (kg/kg) | -22.578 | -0.150 | (-57.837, 12.682) | 17.684 | 0.206 | -0.106 | -16.023 | (-49.304, 17.258) | 16.678 | 0.340 |
|  | | Arm Curl (reps/kg) | -4.595 | -0.027 | (-45.522, 36.332) | 20.515 | 0.823 | 0.100 | 17.152 | (-23.528, 57.831) | 20.375 | 0.403 |
|  | | Chair Stand (reps/kg) | -56.213 | -0.231 | (-112.714, 0.288) | 28.329 | 0.051 | -0.082 | -19.986 | (-77.380, 37.408) | 28.754 | 0.489 |
|  | | Standing Long Jump (cm/kg) | -6.084 | -0.223 | (-12.909, 0.741) | 3.413 | 0.080 | -0.073 | -1.994 | (-9.448, 5.460) | 3.725 | 0.595 |
|  | |  |  |  |  |  |  |  |  |  |  |  |
| DEA | | Handgrip (kg/kg) | -0.032 | -0.178 | (-0.072, 0.009) | 0.020 | 0.124 | -0.151 | -0.028 | (-0.070, 0.014) | 0.021 | 0.192 |
|  | | Arm Curl (reps/kg) | -0.031 | -0.138 | (-0.082, 0.021) | 0.026 | 0.241 | -0.096 | -0.022 | (-0.077, 0.034) | 0.028 | 0.440 |
|  | | Chair Stand (reps/kg) | -0.052 | -0.174 | (-0.121, 0.017) | 0.035 | 0.136 | -0.018 | -0.2e^-3^ | (-0.002, 0.002) | 0.001 | 0.887 |
|  | | Standing Long Jump (cm/kg) | **-0.010** | **-0.292** | **(-0.018, -0.002)** | **0.004** | **0.017** | **-0.360** | **-0.012** | **(-0.021, -0.003)** | **0.004** | **0.007** |
|  | |  |  |  |  |  |  |  |  |  |  |  |
| DHEA | | Handgrip (kg/kg) | 0.0161 | 0.140 | (-0.262, 0.294) | 0.014 | 0.908 | 0.084 | 0.044 | (-0.223, 0.312) | 0.134 | 0.742 |
|  | | Arm Curl (reps/kg) | 0.126 | 0.084 | (-0.223, 0.476) | 0.175 | 0.474 | 0.229 | 0.344 | (-0.015, 0.703) | 0.180 | 0.060 |
|  | | Chair Stand (reps/kg) | 0.053 | 0.026 | (-0.418, 0.525) | 0.237 | 0.823 | 0.184 | 0.371 | (-0.124, 0.866) | 0.248 | 0.139 |
|  | | Standing Long Jump (cm/kg) | -0.019 | -0.083 | (-0.075, 0 .037) | 0.028 | 0.507 | 0.066 | .015 | (-0.047, 0.077) | 0.031 | 0.629 |
|  | |  |  |  |  |  |  |  |  |  |  |  |
| LEA | | Handgrip (kg/kg) | -0.390 | -0.199 | (-0.835, 0.054) | 0.223 | 0.084 | -0.130 | -0.262 | (-0.718, 0.194) | 0.228 | 0.256 |
|  | | Arm Curl (reps/kg) | -0.381 | -0.157 | (-0.945, 0.182) | 0.283 | 0.182 | -0.139 | -0.331 | (-0.891, 0.229) | 0.280 | 0.242 |
|  | | Chair Stand (reps/kg) | -0.205 | -0.062 | (-0.972, 0.561) | 0.384 | 0.595 | -0.120 | -0.393 | (-1.157, 0.371) | 0.383 | 0.308 |
|  | | Standing Long Jump (cm/kg) | **-0.098** | **-0.268** | **(-0.187, -0.010)** | **0.044** | **0.029** | -0.225 | -0.085 | (-0.174, 0.003) | 0.044 | 0.059 |
|  | |  |  |  |  |  |  |  |  |  |  |  |
| SEA | | Handgrip (kg/kg) | -0.325 | -0.157 | (-0.800, 0.149) | 0.238 | 0.176 | -0.107 | -0.231 | (-0.689, 0.226) | 0.229 | 0.317 |
|  | | Arm Curl (reps/kg) | -0.239 | -0.093 | (-0.836, 0.359) | 0.300 | 0.429 | -0.012 | -0.029 | (-0.590, 0.531) | 0.280 | 0.917 |
|  | | Chair Stand (reps/kg) | -0.560 | -0.161 | (-1.364, 0 .242) | 0.403 | 0.168 | -0.129 | -0.450 | (-1.172, 0.272) | 0.362 | 0.218 |
|  | | Standing Long Jump (cm/kg) | **-0.128** | **-0.326** | **(-0.222, -0.035)** | **0.047** | **0.008** | **-0.276** | **-0.113** | **(-0.202, -0.023)** | **0.045** | **0.014** |
| 2-AG: 2-Arachidonoylglycerol; AEA: Anandamida; OEA: Oleoylethanolamide; 2-LG: 2-Linoleoylglycerol; 2-OG: 2-Oleoylglycerol; DEA: Docosatetraenoylethanolamide; LEA: Linoleoylethanolamide; SEA: Stearoylethanolamide; DHEA: Docosahexaenoylethanolamide  Data are presented for all sample as standardized regression coefficient (β), regression coefficient (b), interval coefficients (95% IC), standard error (SE), and P values from the model.  Model 1: Unadjusted; Model 2: Adjusted for age and diabetes for 2-AG, 2-LG, 2-OG, DEA and DHEA or antidepressant medication and diabetes for AEA, OEA, LEA and SEA | | | | | | | | | | | | |
